# COPD – do the right thing

**DOI:** 10.1186/s12875-021-01583-w

**Published:** 2021-12-11

**Authors:** Hanna Sandelowsky, Ulla Møller Weinreich, Bernt B. Aarli, Josefin Sundh, Kristian Høines, Georgios Stratelis, Anders Løkke, Christer Janson, Christian Jensen, Kjell Larsson

**Affiliations:** 1 Department of Medicine, Clinical Epidemiology Division T2, Karolinska University Hospital, Karolinska Institutet, Solna, SE-171 76 Stockholm, Sweden; 2 Division of Family Medicine and Primary Care, Department of Neurobiology, Care Sciences and Society, Karolinska Institutet, Stockholm, Sweden; 3 Academic Primary Healthcare Centre, Stockholm County Stockholm, Sweden; 4 Department of Respiratory Diseases, Aalborg University Hospital, Aalborg, Denmark; 5 The Clinical Institute, Aalborg University, Aalborg, Denmark; 6 Department of Clinical Science, University of Bergen, Bergen, Norway; 7 Department of Thoracic Medicine, Haukeland University Hospital, Bergen, Norway; 8 Department of Respiratory Medicine, Faculty of Medicine and Health, Örebro University, Örebro, Sweden; 9 Tananger Medical Center, Tananger, Norway; 10 Department of Medical Sciences: Respiratory, Allergy and Sleep Research, Uppsala University, Uppsala, Sweden; 11 AstraZeneca Nordic, Södertälje, Sweden; 12 Department of Medicine, Little Belt Hospital, Vejle, Denmark; 13 Department of Regional Health Research, University of Southern Denmark, Odense, Denmark; 14 Lægehuset Remisen, Præstø, Denmark; 15 Integrative Toxicology, National Institute of Environmental Medicine, IMM, Karolinska Institutet, Stockholm, Sweden

**Keywords:** Pulmonary disease, Chronic obstructive, Primary health care, Referral and consultation general practice

## Abstract

A gap exists between guidelines and real-world clinical practice for the management and treatment of chronic obstructive pulmonary disease (COPD). Although this has narrowed in the last decade, there is room for improvement in detection rates, treatment choices and disease monitoring. In practical terms, primary care practitioners need to become aware of the huge impact of COPD on patients, have non-judgemental views of smoking and of COPD as a chronic disease, use a holistic consultation approach and actively motivate patients to adhere to treatment.

This article is based on discussions at a virtual meeting of leading Nordic experts in COPD (the authors) who were developing an educational programme for COPD primary care in the Nordic region. The article aims to describe the diagnosis and lifelong management cycle of COPD, with a strong focus on providing a hands-on, practical approach for medical professionals to optimise patient outcomes in COPD primary care.

## Background

Approximately 384 million people worldwide have chronic obstructive pulmonary disease (COPD) [1], and it is estimated that over half of patients with COPD may be undiagnosed [2]. According the World Health Organization, COPD is the third leading cause of death globally [3] and costs are estimated to be in excess of 100 billion dollars per year [4, 5]. Most patients with COPD are fully managed in primary care [6, 7]. As the first-line healthcare provider, and often the gatekeeper to secondary care, primary care is not only responsible for the early detection of chronic diseases like COPD, but it also has a unique opportunity to provide patients with evidence-based treatments. Currently, there is a gap between COPD guidelines and real-world clinical practice, and although this has narrowed in the last decade [8, 9], there is room for improvement in detection rates, treatment choices and disease monitoring [8, 10–14]. This article is based on discussions that took place during a virtual meeting on 15 June 2020 involving leading Nordic experts in COPD (the authors). The purpose of the meeting was to finalise a COPD educational programme that had been developed for primary care in the Nordic region. During the course of discussions, the authors agreed there was a scientific need to author this opinion piece, with a strong emphasis on providing a hands-on, practical approach for General Practice. Thus, we discuss the burden of disease, the impact of symptoms and exacerbations and the unmet needs in the lifelong clinical management cycle of COPD.

## Main text

### Burden of disease

COPD is a heterogeneous disease characterised by persistent respiratory symptoms and airflow limitation [1]. It provides a significant burden for patients and society due to the natural progression of COPD, reduced daily/physical activities, exacerbations and impact on work and social isolation, which often leads to psychological conditions such as anxiety and depression [1, 15, 16]. Together, these factors decrease health-related quality of life (HRQL) and increase mortality, compared with the general population [1], and are further confounded by a number of comorbidities that are frequently associated with COPD, including cardiovascular, metabolic and other chronic conditions (see the ‘Common comorbidities’ section for additional details [17, 18]).

### How does the disease manifest?

Symptoms of COPD may include chronic and progressive dyspnoea, chronic cough, sputum production, chest tightness or fatigue [1, 19–21]. In many patients, chronic cough with or without sputum production may be intermittent and may precede the development of airflow limitation by many years [1, 22]. Indeed, the occurrence of a productive cough in young smokers constitutes a substantial risk of developing COPD in later life [23]. Conversely, dyspnoea and decline in health status are weakly correlated with airflow limitation [19, 21]. As a result of this variation, symptoms may not be obvious until forced expiratory volume in 1 s (FEV_1_) has declined to approximately 50–60% of predicted, therefore COPD typically remains undiagnosed until it has reached at least moderate severity [24]. COPD should therefore be considered in any patient with a history of exposure to risk factors. Patients with COPD may also be more prone to ‘winter colds’ or ‘acute bronchitis’, and primary care practitioners should consider the possibility of COPD in middle-aged or older smokers with recurrent respiratory tract infections [1, 25, 26].

#### Dyspnoea is a key everyday modality

Dyspnoea due to airflow limitation is the main symptom of COPD and is often the reason why patients with COPD seek medical care [19]. Dyspnoea is a problem not only in patients with severe disease, but also in patients with mild-to-moderate disease, where symptoms may only be evident during exercise [27, 28]. Milder symptoms may be dismissed by the patient as being related to age, general fitness or other factors [29], and patients typically do not seek medical help until symptoms have a substantial impact on their daily life [1, 30].

**Fig. 1:**
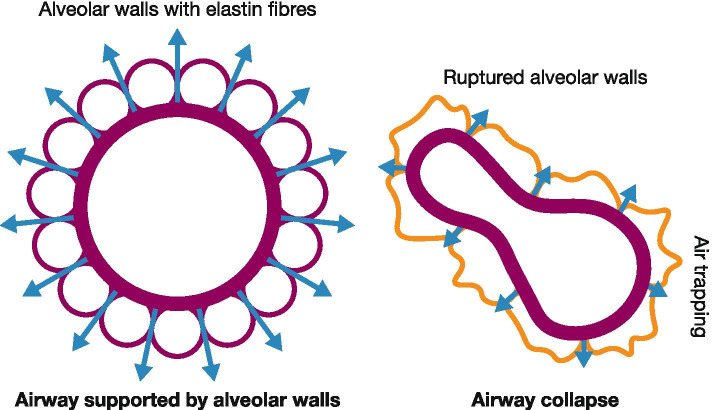
The pathophysiological background to dyspnoea. Alveolar wall destruction leads to loss of elastic ‘passive’ recoil of the alveoli and support for the bronchiole is lost, leading to bronchiole collapse, which in turn results in air trapping, static hyperinflation and increased functional residual capacity [32, 41–43]

The cause of dyspnoea is multifactorial but is dominated by parenchymal destruction and loss of elastic recoil, which leads to the collapse of the small airways during expiration and thus longer expiration times (Fig. 1). As the respiratory rate increases during exercise, loss of elastic recoil means that patients may not be able to exhale completely before their next breath [31]. This, in turn, leads to a gradual increase in end-expiratory lung volume, resulting in ‘dynamic hyperinflation’, which contributes considerably to the sensation of dyspnoea during exercise [32–34] and leads to reduced daily physical activity [35]. Luminal obstruction due to mucus plugging and increased airway wall thickness may further aggravate dyspnoea [36, 37]. Furthermore, the severity of dyspnoea [38, 39] along with the magnitude of hyperinflation [40] are both strong predictors of mortality.

Sarcopenia, which is common in COPD, adds to the impact of dyspnoea due to reduced muscle mass leading to decreased exercise capacity and increased breathlessness [44]. Heart failure is also a common comorbidity in patients with COPD and can contribute to a sensation of dyspnoea [17, 45].

Symptoms vary by day and season. Daily symptoms are typically bimodal in distribution, being particularly troublesome in the early morning and in the evening/at night-time, while seasonal variation in symptoms leads to a peak in exacerbations through autumn and winter [46–48].

Previous studies have found that 37% of all patients and 46% of patients with severe COPD reported worse symptoms in the morning than at any other time of day [47], and morning symptoms have been associated with general physical impairment [49]. Similarly, 46% of all patients and 61% of patients with severe COPD have reported evening/night-time symptoms [47, 48]. Singh et al. found that nearly two-thirds of patients experienced night-time symptoms, regardless of disease severity [50]. During the night, coughing was the most prevalent symptom, reported in 43% of patients; bringing up phlegm, dyspnoea and wheezing were also reported in around one-third of patients. Night-time symptoms significantly impact sleep quality [50], which may lead to exhaustion and further physical decline.

In a study by Partridge et al., although many patients with COPD regarded respiratory symptoms during morning routines as bothersome, most patients were not taking their COPD medication in time for it to exert its effect [47]. This suggests that physicians should actively question patients about their morning activities and evening/night-time symptoms to fully assess disease impact and optimise the timing of treatment [47].

### Causes and drivers of COPD

According to the classical Fletcher–Peto model [51], FEV_1_ decreases gradually over a lifetime. In susceptible individuals exposed to smoke or other noxious gases who develop COPD, the decline progresses more rapidly. However, the rate of lung function decline in COPD is highly variable, being negatively affected by smoking and exacerbations, but also remains relatively stable for long periods in many patients [52]. Increased incidence of COPD has been associated with indoor and outdoor air pollution [53] (particularly the burning of biomass/solid fuels in developing countries and rural areas [54, 55]), occupational exposure to dust and gases [56, 57] and lower socioeconomic status and poverty [1, 58]; other risk factors include genetic predisposition, abnormal lung development and accelerated ageing [1].

One major driver of the disease process in COPD is ongoing chronic inflammation of the small airways, known as bronchiolitis [59]. Neutrophilic granulocytes, CD8^+^ lymphocytes and macrophages were initially identified as being among the most important in the pathogenesis of COPD [60], but we now know that other immune cells, such as eosinophils, are also involved [61]. Recruitment and activation of immune cells and release of inflammatory mediators lead to mucous hypersecretion, luminal obstruction/mucous plugging and tissue destruction [37, 41]. Inflammation is also coupled with a dysfunctional repair/remodelling process that destroys the alveolar walls and thickens the walls of the airways [37, 42]. Pulmonary vascular remodelling may also result in pulmonary hypertension and right-sided heart failure (cor pulmonale) [62]. Bronchiectasis may also add to the symptom burden [63].

### Exacerbations

COPD is also characterised by periodic acute worsening of symptoms beyond normal day-to-day variation, known as acute exacerbations; symptoms can last several weeks, and severe exacerbations result in hospitalisation and increased risk of death (Table 1) [1, 64].

**Table 1:** Classification of exacerbations

	Treatment classification	Clinical classification	Physical assessment
**Mild**	Treated with short-acting bronchodilators only (patient self-management)	Does not involve respiratory failure	Dyspnoea ranging from insignificant to troublesome Respiratory rate < 25 breaths/minute Heart rate < 110 beats/minute ≥90% oxygen saturation
**Moderate**	Treated with antibiotics and/or oral steroids	Acute respiratory failure, non-life-threatening	
**Severe**	Emergency room visit or hospitalisation	Acute respiratory failure, life-threatening	Cyanosis or oedema could be evident Respiratory rate > 25 breaths/minute, Heart rate > 110 beats/min < 90% oxygen saturation PaO_2_ < 8.0 kPa PaCO_2_ < 6.5 kPa
**Life-threatening**	Hospitalisation	Acute respiratory failure, life-threatening	May be confused/unconscious Variable respiratory rate Variable heart rate < 90% oxygen saturation PaO_2_ < 6.5 kPa PaCO_2_ > 9.0 kPa Blood gas pH < 7.3

From references [1, 65–67]

*PaCO*_*2*_ partial pressure of carbon dioxide, *PaO*_*2*_ partial pressure of oxygen

**Fig. 2:**
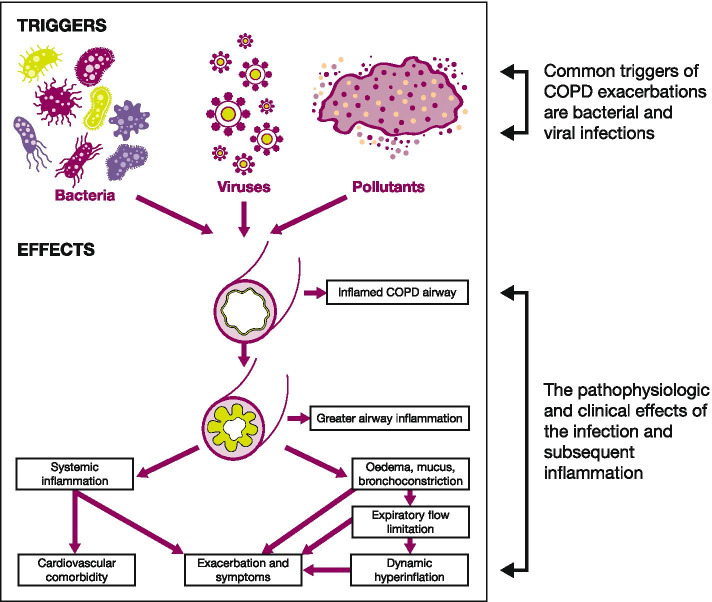
The pathophysiology of COPD exacerbations. Reproduced with permission from: Wedzicha JA, Seemungal TAR. COPD exacerbations: defining their cause and prevention. Lancet. 2007;370(9589):786–96. Copyright © Elsevier 2007. *COPD* chronic obstructive pulmonary disease

Exacerbations are characterised by an initial increase in airway inflammation, resulting in airway oedema, mucus production and bronchoconstriction (Fig. 2) [68]. An acute exacerbation is a serious event triggering a catastrophic cascade that is potentially overwhelming and life-threatening, akin to a ‘stroke of the lungs’ [69]. There is also emerging evidence showing that a proportion of patients with mild COPD may be susceptible to exacerbations [70]; however, at present, due to their mild disease, these patients might not be identified for intervention [71]. It is vital to improve early detection and diagnosis of patients with mild COPD in primary care. Smoking cessation and/or recognising harmful work exposures early may halt disease progression, and simple measures may be taken to reduce the risk of exacerbations, such as influenza and pneumococcal vaccinations.

**Fig. 3:**
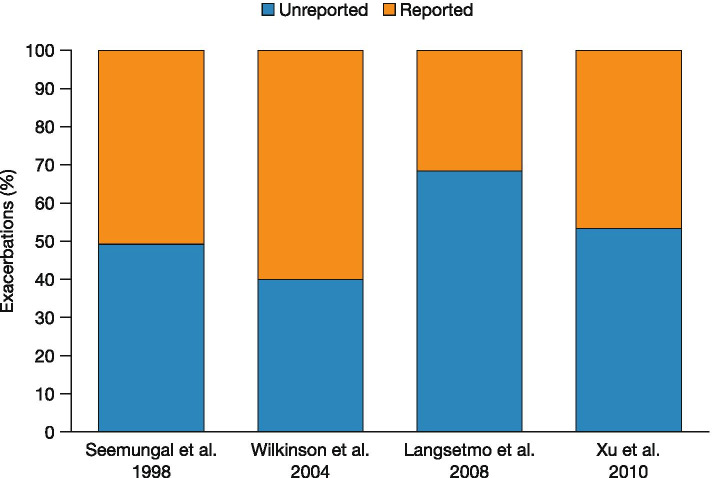
Proportion of exacerbations under-reported in different studies. From references [76, 98–100]

COPD exacerbations are associated with a more rapid decline in FEV_1_ [71–74], poorer HRQL [71, 75–77], hospitalisations [1, 72], increased risk of cardiovascular disease (CVD) [78–81], risk of further exacerbations [82] and increased mortality [1, 39, 82–87]. Moreover, the risk of severe exacerbation or death has been found to increase with each subsequent exacerbation [82]. Even a single exacerbation can drastically decrease HRQL (St George’s Respiratory Questionnaire total score) [75, 88], with HRQL remaining worse 6 months later in some patients, versus no exacerbations [89]. In addition, damage from exacerbations has been found to go beyond the lungs due to the associated systemic inflammation during the exacerbation [68] and may increase the risk of thromboembolism, stroke, myocardial infarction and cardiovascular mortality, particularly following a severe exacerbation [78, 90–92]. While in around 30% of exacerbation cases, no clear cause is found [93], exacerbations are known to be triggered by multiple factors, including infections and exposure to particulate matter [1, 94]. In patients with moderate/severe COPD, bacterial pathogens account for 40–50%, viral pathogens for 30–40% and atypical bacteria for 5–10% of exacerbations [94]; following an exacerbation, bacterial colonisation has been found to rise to 70%, with *Haemophilus influenzae*, *Streptococcus pneumoniae* and *Moraxella catarrhalis* being the most common species found [95]. While many patients take some action (e.g. rest, cut down on smoking, increase medication etc.), over 40% of patients do not contact healthcare providers during an exacerbation [96]. Under-treatment is also common in patients hospitalised because of severe COPD exacerbations [97]. Across four different studies, between 40 and 68% of exacerbations were under-recognised, under-reported and thus under-treated; moreover, this proportion was largely unchanged over 12 years (Fig. 3) [76, 98–100]. This suggests that patients with COPD require education on exacerbation symptoms and when to seek professional healthcare [101].

Several factors may predict risk of future exacerbations (Table 2) and should be considered when initiating treatment and at follow-up/annual review.

**Table 2:** Summary of predictors of future exacerbations

Predictors of future exacerbations	
**• Smoking**	
**• A history of previous exacerbations**	
**• Blood eosinophil count**	
**• CAT score ≥ 20**	
**• Presence of chronic bronchitis**	
**• Comorbidities**	
**• Severity of airflow limitation**	

From references [102–107]

*CAT* COPD assessment test, *COPD* chronic obstructive pulmonary disease

Elevated blood eosinophils are associated with higher COPD-related hospital readmission rates and all-cause readmission [108] (see the ‘Pharmacological treatment of COPD’ section).

### Diagnosis of COPD

COPD should be considered in any patient with clinical symptoms and risk factors for the disease (predominantly smoking). Diagnosis is confirmed by a post-bronchodilator FEV_1_/forced vital capacity (FVC) ratio < 0.70 [1] or below the lower limit of normal. Of note, the FEV_1_/FVC ratio has a natural decline with age, and the fixed ratio may lead to an underestimation of COPD in younger patients and an overestimation in elderly patients [109–111].

Spirometry is important not only for the diagnosis of COPD, but also to classify the severity of airflow limitation (based on FEV_1_% predicted) [1]. In addition, baseline and post-baseline FEV_1_ measurements can be compared with future spirometric assessments to identify patients who demonstrate a rapid decline in lung function (rapid decliners), and may be used when considering alternative diagnoses, such as when symptoms appear disproportionate to the degree of airflow obstruction [1]. It is important to ensure that healthcare professionals first carry out spirometry [112] and that those who carry out spirometry are appropriately trained [113], as incorrect interpretation or performance of the procedure readily leads to misdiagnosis of COPD [114].

Reversibility with a fast-acting bronchodilator has historically been used to distinguish asthma from COPD; however, it is now accepted that significant reversibility (FEV_1_ ≥ 12% and ≥ 200 mL change from baseline) or FVC ≥10% is at least as common in patients with COPD, despite having no features of asthma [115–118], and can vary in the same patient between assessments for both asthma and COPD [119, 120]. A significantly improved FEV_1_ with normalisation of the FEV_1_/FVC ratio post-bronchodilator or post 4–12 weeks of inhaled corticosteroids (ICS) means that COPD, but not asthma, can be excluded.

Once confirmed as COPD, phenotyping is important to ensure optimal pharmacological and non-pharmacological treatment [1]. Typically, patients with COPD and low eosinophil counts have a poor response to ICS while patients with higher levels of eosinophils or asthma–COPD overlap have a better response to ICS [1]. Another phenotype of importance, with regard to medication, is the frequent exacerbator, defined as patients with ≥2 exacerbations per year [121]. Other phenotypes described are chronic bronchitis, emphysematous and rare exacerbator; emerging COPD phenotypes included pulmonary cachexia phenotype, overlap COPD and bronchiectasis, upper lobe-predominant emphysema phenotype, rapid decliner, comorbidities or systemic phenotype, α-_1_ antitrypsin deficiency and no-smoking COPD [122].

COPD is often overlooked in the initial consultation between physician and patient. Some patients with COPD appear to be asymptomatic, adapt to symptoms and adjust their daily activities to reduce their occurrence [29]; thus, it is vital that patients are asked the correct questions during the consultation with their physician so that as much information can be obtained as possible. It is only by understanding the impact of COPD on multi-morbidities or other health issues that a patient can be optimally prioritised in primary healthcare [123]. Furthermore, in order to maximise the window of opportunity to impact disease progression (e.g. via smoking cessation), it is necessary to be proactive and diagnose early during the mild disease stage [124].

### COPD severity and characterisation by the ABCD assessment tool

**Fig. 4:**
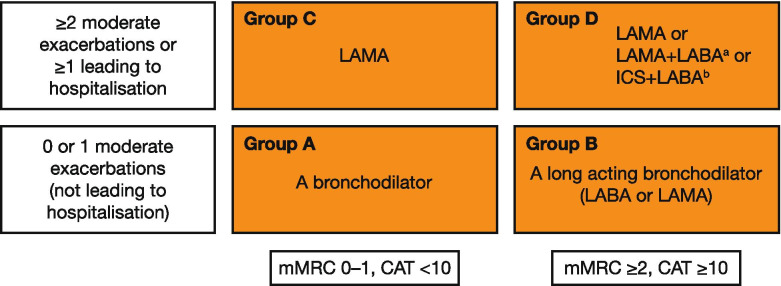
Disease characteristics and initial pharmacological treatment algorithm. ^a^Consider if highly symptomatic (e.g. CAT > 20). ^b^Consider if EOS ≥0.3 × 10^9^ cells/L (≥300 cells/mm^3^). Reproduced with permission from: Global Initiative for Chronic Obstructive Lung Disease 2021 Report; Figure 4.2. Available from https://goldcopd.org/wp-content/uploads/2020/11/GOLD-REPORT-2021-v1.0-16Nov20_WMV.pdf, accessed 18 November 2020. *CAT* COPD assessment test, *COPD* chronic obstructive pulmonary disease, *EOS* eosinophil count, *ICS* inhaled corticosteroids, *LABA* long-acting β_2_-agonist, *LAMA* long-acting muscarinic antagonist, *mMRC* modified Medical Research Council dyspnoea scale

Whereas the Global Initiative for Chronic Obstructive Lung Disease (GOLD) once focussed primarily on airflow limitation as a measure of COPD severity (GOLD stages 1–4 – mild-to-very-severe COPD) to characterise patients, the ABCD assessment tool adds patient symptoms (based on the COPD assessment test [CAT] and/or modified Medical Research Council dyspnoea scale [mMRC]) and exacerbation history in the previous 12 months to the assessment (Fig. 4) [1]. Patients in Group A have a low risk of future exacerbations with low symptom burden; patients in Group B have a low risk of future exacerbations but significant symptom burden; patients in Group C have a high risk of future exacerbations and low disease burden; patients in Group D have a high risk of future exacerbations and significant disease burden.

In addition to the ABCD assessment, the 6-min walking test is a simple but very useful tool for assessing impaired exercise tolerance [125]; in patients who have experienced a severe exacerbation, lower physical activity levels are associated with increased all-cause mortality [126].

### Differential diagnoses – could it be something else?

Diagnosing COPD can sometimes be challenging and, particularly as it is a lifelong diagnosis, it is of utmost importance that the clinician gives full consideration to alternative diagnoses and is aware of the many pitfalls and potential impact of misdiagnosis of COPD. Clinical history, patient characteristics and exposure are important considerations, in addition to spirometry, to differentiate not only between obstructive lung diseases (asthma, bronchiectasis and COPD) but also restrictive lung disease and heart failure, as these may both be misinterpreted as COPD or may co-exist with COPD (Table 3) [1, 127, 128].

**Table 3:** Main differential diagnoses of COPD

Diagnosis	Characteristics
**COPD**	• Onset in mid-life and later
	• Symptoms slowly progressive
	• History of smoking or exposure to other types of pollution
**Asthma**	• Onset usually early in life
	• Symptoms vary widely from day to day
	• Symptoms worse at night/early morning
	• Allergy, rhinitis and/or eczema also present
	• Family history of asthma
	• Obesity can co-exist
**Congestive heart failure**	• Chest X-ray shows dilated heart
	• Older patients
	• Symptoms slowly progressive
	• Pulmonary oedema or ankle swelling
	• Night-time symptoms (orthopnoea)
	• Pulmonary function tests indicate volume restriction, not airflow limitation
**Bronchiectasis**	• No or sparse smoking history
	• Large volumes of purulent sputum
	• Frequent exacerbations
	• Possible co-existence of auto-immune disease
	• Commonly associated with bacterial infection
	• Chest X-ray shows bronchial dilation/wall thickening

Reproduced with permission from: Global Initiative for Chronic Obstructive Lung Disease 2021 Report; Table 2.7. Available from https://goldcopd.org/wp-content/uploads/2020/11/GOLD-REPORT-2021-v1.0-16Nov20_WMV.pdf, accessed 18 November 2020

*COPD* Chronic obstructive pulmonary disease

Other useful diagnostic tools include blood tests for haemoglobin, erythrocyte sedimentation rate, eosinophil count and N-terminal pro B-type natriuretic peptide (NT-proBNP). Electrocardiograms, chest X-rays and echocardiography in patients with increased NT-proBNP may detect the presence of significant comorbidities, such as CVD, heart failure, lung cancer, pleural disease and changes associated with COPD [1, 129–131].

Computed tomography (CT) or high-resolution CT can be helpful for quantifying emphysema and for the differential diagnosis of bronchiectasis, suspected lung cancer or interstitial lung disease [1, 130]. Pulmonary lung function can further be evaluated by measuring the lung volumes in a plethysmograph and by measuring the diffusion capacity of the lungs for carbon monoxide (DLCO). DLCO may be helpful to differentiate between asthma (usually normal or increased DLCO) and mild COPD (impaired DLCO due to emphysema) but is not a routine examination [1, 132, 133].

#### Differential diagnoses of exacerbations

As the symptoms during an exacerbation are not specific to COPD worsening, relevant differential diagnoses should be considered e.g. pneumonia, pneumothorax, pulmonary embolism, cardiac arrhythmias or heart failure (Table 3) [134, 135]. Patients with COPD experiencing pneumonia typically present with very similar symptoms as patients experiencing an COPD exacerbation associated with infection [1]; however, while treatment is generally similar, the duration of antibiotic treatment for pneumonia can be longer in practice [136].

### Common comorbidities

COPD often co-exists with other diseases that may have a significant impact on prognosis [17, 18, 137, 138]. Indeed, it has been suggested that almost all patients have ≥1 comorbidity, with half having ≥4 [139, 140]. Some comorbidities arise independently of COPD, whereas others are causally related, either with shared risk factors (e.g. age, smoking, systemic inflammation) or by one disease increasing the risk severity of the other [18, 141]. Common comorbidities include bronchiectasis, CVD, chronic kidney disease, dyslipidaemia, diabetes, hypertension, lung cancer, mental disorders, osteoporosis, obstructive sleep apnoea syndrome and skeletal muscle dysfunction [17, 18, 137]. Ellingsen et al. found that the strongest predictor of death in patients with COPD was comorbid heart failure, which was associated with a nearly doubled mortality risk [142]. Stroke and myocardial infarction were also associated with an approximately 50% increased risk of death. An observational retrospective registry study of > 21,000 patients with diagnosed COPD found that the frequency of comorbidities increased during 8 years of observation (Table 4) [8].

**Table 4:** Common comorbidities of COPD [8]

Comorbidity	At diagnosis (%)	During 8-year period after diagnosis (%)^**a**^
**Hypertension**	33.7	51.4
**Asthma**^**b**^	23	
**Pneumonia**	24.6	42.9
**GERD**^**c**^	17–54	
**Ischaemic heart disease**	15.6	24.3
**Heart failure**	15.2	31.4
**Depression**	11.8	20.2
**Diabetes**	11.6	18.8
**Myocardial infarction**	9.0	17.0
**Osteoporosis**	7.6	16.9
**Stroke**	5.8	12.3
**Lung cancer**	1.0	4.6

*COPD* Chronic obstructive pulmonary disease, *GERD* Gastro-oesophageal reflux disease

^a^Cumulative data

^b^Following diagnosis of COPD

^c^Prevalence among all patients diagnosed with COPD [143]

Due to the significant impact of comorbidities on prognosis and the rising incidence with time and disease progression [8, 141, 143], it is vital that comorbidities are assessed at diagnosis and at every follow-up review (Table 4). In addition, since the psychological impact of COPD (e.g. anxiety and depression) is known to be substantial [144, 145] and is associated with higher mortality [146], it is important that psychological health is assessed at diagnosis.

### Treatment of COPD

COPD treatment goals include smoking cessation (or the termination of other exposure), symptom relief (improved physical capacity and reduced dyspnoea) and reduced risk of exacerbations and mortality [1]. The treatment approach for COPD should be based on clinical, functional and/or biological features that can be observed at the individual level, i.e. treat the treatable traits. Management options include pharmacological and non-pharmacological treatments carried out by a multidisciplinary team comprising a physician, specialist nurse and physiotherapist, as well as a counsellor/psychologist, occupational therapist and nutritionist in some cases [1, 147].

#### Pharmacological treatment of COPD

Most current therapies target the physiological changes and associated symptoms that result from and aim to improve airflow by altering smooth muscle tone (bronchodilators) or suppressing inflammation (corticosteroids, roflumilast), leading to improved HRQL and symptom control, while reducing the risk of exacerbations and mortality [1].

Initial pharmacological treatment is guided by use of the GOLD ABCD assessment tool at the time of diagnosis and depends on the symptom burden measured by CAT or mMRC, exacerbation history, blood eosinophil count and the presence of co-existing asthma (Fig. 4). Bronchodilators form the mainstay of symptomatic COPD therapy. These come in the form of short-acting β_2_-agonists and long-acting β_2_-agonists (LABAs) and short-acting muscarinic antagonists and long-acting muscarinic antagonists (LAMAs) [1]. Short-acting bronchodilators have typically been used as rescue medication; however, guidelines now recommend maintenance treatment with long-acting bronchodilators as a more effective means of reducing symptoms with an additional effect of preventing exacerbations [1]. When LAMAs and LABAs are combined, their complementary mechanisms of action provide greater improvements in lung function and greater reductions in symptoms than the respective monotherapies [1, 148].

There is strong evidence to support the use of ICS in combination with a LABA or LABA/LAMA (‘triple therapy’) in patients with a history of ≥2 moderate exacerbations in the previous year, ≥1 hospitalisation for COPD exacerbations, blood eosinophils ≥0.3 × 10^9^ cells/L (≥300 cells/mm^3^) or a history of/or concomitant asthma [1, 65, 149–151]. GOLD also recommends that the use of ICS in combination with a long-acting bronchodilator be considered in patients with one exacerbation during the previous year or when blood eosinophils are 0.1–0.3 × 109 cells/L (100–300 cells/mm^3^) [1]. Combining an ICS with a LABA or with a LAMA/LABA combination has been shown to reduce exacerbations to a greater extent than bronchodilators alone [149, 151–153]. Recent clinical trials in symptomatic patients with COPD and a history of exacerbations, despite maintenance therapy, found that treatment with fixed-dose ICS/LABA/LAMA triple therapy significantly lowered rates of moderate or severe COPD exacerbations, improved lung function, reduced symptoms and improved HRQL versus treatment with LABA/LAMA or ICS/LABA [65, 150, 151].

Triple therapy has also been shown to reduce exacerbations versus LABA/LAMA in symptomatic patients with no exacerbations or only one exacerbation in the previous 12 months (i.e. patients who would be defined as GOLD Group B) [149, 154]. The GOLD recommendations around eosinophil counts are informed by evidence that the treatment effect of ICS is influenced by blood eosinophil levels, with the benefit generally increasing at eosinophil counts > 0.1 × 10^9^ cells/L (100 cells/mm^3^) [1]. A pooled analysis of three clinical trials showed a 25% risk reduction in exacerbations with the addition of ICS at eosinophil counts of > 0.1 × 10^9^ cells/L, which increased to 50% at eosinophil counts of > 0.34 × 10^9^ cells/L (340 cells/mm^3^) [152].

The use of oral prophylactic macrolide antibiotics has been shown to reduce the risk of COPD exacerbations [155, 156]. For this reason, the GOLD guidelines suggest macrolide antibiotics as a possible add-on treatment for frequent exacerbators. However, long-term macrolide use remains controversial due to the risk of bacterial resistance and hearing test impairments [1].

##### Mortality

Mortality is the ultimate outcome in COPD and risk of mortality can be reduced in several ways: smoking cessation, reduced frequency and severity of exacerbations, lung rehabilitation, increased physical activity and pharmacological treatment [1]. Long-term oxygen therapy has also long been known to decrease mortality in patients with persistent hypoxia [157, 158]. In addition to oxygen therapy, non-invasive ventilation has been found to prolong the time to readmission or death and may benefit some patients with COPD [159].

Randomised trials and retrospective cohort analyses have suggested a benefit for survival with ICS-containing therapy in high-risk patients with COPD compared with placebo or non-ICS therapy [160–163]. Indeed, the TORCH study demonstrated a 17.5% reduction of risk of death with a borderline significance (*p* = 0.052) in favour of ICS/LABA treatment versus placebo [160].

Treatment with ICS/LABA/LAMA triple therapy has been demonstrated to significantly reduce the rate of moderate-to-severe exacerbations and improve lung function, symptoms and HRQL compared with LABA/LAMA and ICS/LABA treatment [65, 149–151]. Thus, triple therapy is currently recommended for patients with insufficient symptom control or exacerbations despite prior treatment with dual therapy treatment [1]. Recently, two large trials of fixed-dose combination treatment with ICS/LABA/LAMA (IMPACT [65] and ETHOS [151]) found that all-cause mortality was reduced in high-risk patients with COPD compared with LABA/LAMA treatment. Moreover, the study by Martinez et al. showed that the risk of death from any cause with triple therapy was 49% lower than that in the LABA/LAMA group [164], with the beneficial effect on mortality attributable to a reduction in cardiovascular death, and the risk reduction in parity with the effects observed in prevention of cardiovascular events observed in coronary heart disease and statin trials [165–168].

Despite these benefits, the use of ICS has been associated with a slightly increased risk of pneumonia [169] and use of ICS is not recommended for patients with repeated prior pneumonia events, mycobacterial infection or blood eosinophils < 0.1 × 10^9^ cells/L (< 100 cells/mm3) [1]. Of note, withdrawal of ICS in patients with higher eosinophil counts increases the risk of exacerbations from 0.15 × 10^9^ cells/L, being most pronounced at ≥0.3 × 10^9^ cells/L [170], a finding that was not corroborated in another study [171]. In addition, according to the current GOLD guidelines, ICS should not be used in patients assessed as being in GOLD Group A or B, i.e. patients who have not yet experienced an exacerbation, unless the patient has asthma as a comorbidity [1].

Current guideline recommendations propose a reactive, stepwise approach for escalating pharmacological treatment of COPD, where a patient must have suffered ≥2 moderate exacerbations or one hospitalisation in the previous year, despite appropriate bronchodilator treatment [1]. This raises the question ‘why?’, since this approach is not applied in other chronic conditions, such as CVD [172]. Physicians must be vigilant in their work and be more ambitious in employing rigorous risk assessment to prevent even the first severe exacerbation leading to hospitalisation since those are associated with increased risk of mortality (Table 2). Indeed, one in five patients with COPD die within 1 year of their first-ever severe exacerbation leading to hospitalisation [86], while half die within 3.6 years of their first-ever severe exacerbation [82].

#### Non-pharmacological treatment of COPD

The risk of exacerbations has been shown to decrease by 22% in ex-smokers compared with current smokers [173], thus smoking cessation is the most important initial step in treatment; advice regarding smoking cessation should be combined with nicotine and non-nicotine replacement therapy to increase long-term smoking abstinence [1, 174]. Pulmonary rehabilitation, including physical activity and self-management education, relieves symptoms and improves HRQL [1, 175]. In addition, vaccinations are also very important: influenza vaccination is recommended for all COPD patients, as it reduces serious illness and death, and pneumococcal vaccination is recommended for patients aged ≥65 years, as it reduces the risk of pneumonia [1]. In malnourished patients with COPD, nutritional support is recommended as it may improve respiratory muscle strength and HRQL.

Reduced physical activity in patients with COPD has been independently linked with reduced HRQL [176], increased hospitalisations [177], physical deconditioning [27, 178] and mortality [179]. Thus, improvement in physical activity and behavioural intervention provides better long-term outcomes for patients with COPD [35, 180, 181].

Finally, long-term oxygen therapy (> 15 h per day) has been shown to improve survival in patients with severe resting hypoxemia [182], but has not been found to be beneficial for patients with stable COPD and moderate resting or exercise-induced arterial desaturation [183].

##### Patient education

In terms of education, it is paramount to explain to patients in plain, understandable language about their diagnosis and prognosis, and to encourage them to be an active participant in their self-management and treatment programme [1]. This will also involve training on how to use their inhaler effectively, as poor technique is a leading cause of poor response to treatment [184, 185]. In addition, patient education should be a shared duty of healthcare systems and wider society, including schools and workplaces. Community engagement alongside health promotion regulations, such as no smoking laws and anti-smoking campaigns, can contribute to disease prevention.

### Follow-up visits to monitor COPD – why and how?

COPD is a complex, progressive disease, therefore routine, individualised monitoring/assessment at follow-up is essential. International and national guidelines suggest annual follow-up visits for stable, non-exacerbating patients at least once per year and a follow-up visit 1–4 weeks after an acute exacerbation for a thorough, overall medical assessment that includes spirometric measurements, documentation of symptom burden and optimisation of COPD medication [1, 186]. Ideally, patients with very severe COPD should be reviewed at least twice a year and multimorbid patients should see their physician at least once a year [186, 187]. The key issue for all members of the multidisciplinary team responsible for monitoring/follow-up is to plan for the next visit based on the patient’s individual needs.

**Fig. 5:**
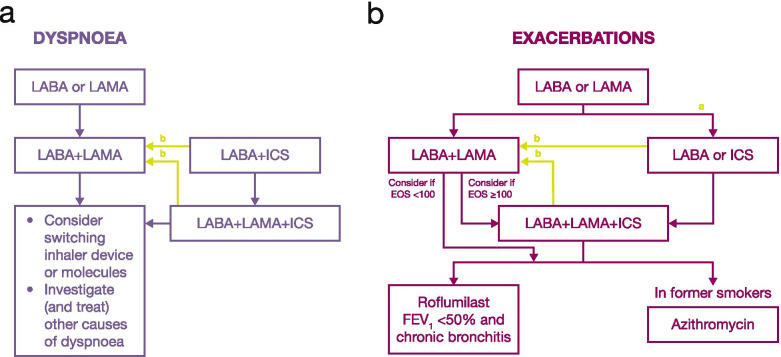
Treatment algorithm for patients with **a)** dyspnoea or **b)** exacerbations at review. ^a^Consider if EOS ≥0.3 × 10^9^ cells/L (≥300 cells/mm^3^) or EOS ≥0.1 × 10^9^ cells/L (≥100 cells/mm^3^) AND ≥ 2 moderate exacerbations/1 hospitalisation. ^b^Consider de-escalation of ICS or switch in response to pneumonia, inappropriate original indication or lack of response to ICS. Reproduced with permission from: Global Initiative for Chronic Obstructive Lung Disease 2021 Report; Figure 4.4. Available from https://goldcopd.org/wp-content/uploads/2020/11/GOLD-REPORT-2021-v1.0-16Nov20_WMV.pdf, accessed 18 November 2020. *EOS* eosinophil count, *FEV*_*1*_ forced expiratory volume in 1 s, *ICS* inhaled corticosteroid, *LABA* long-acting β_2_-agonist, *LAMA* long-acting muscarinic antagonist

Recent GOLD guidelines have included a pharmacological follow-up algorithm that can be applied to any patient who is already taking maintenance treatment(s) irrespective of the GOLD group allocated at treatment initiation (Fig. 5) [1]. At follow-up/annual review, the first priority is to identify whether the initial treatment was successful in helping the patients meet their treatment goals, whether disease progression has occurred and what the predominant symptom is (dyspnoea, exacerbations or both). If dyspnoea dominates, treatment should be stepped up to dual bronchodilator therapy or triple therapy depending on the patients’ current pharmacological maintenance treatment (Fig. 5a). If exacerbations dominate (or both dyspnoea and exacerbations), the alternative algorithm should be followed (Fig. 5b). In addition, patient handling of the inhaler and inhalation technique must be reviewed both annually and before stepping up medication [184, 185, 188]. Recommendations for the annual review are shown in Table 5.

**Table 5:** Recommendations for the review of patients with COPD^a^

Review	Discuss and encourage	Consider
**• The effect of treatment**	• Smoking cessation/continued cessation	• Comorbidities
**• Symptoms (CAT/mMRC/6-min walking test)**	• Medication adherence	• Referral to pulmonary rehabilitation
**• Exacerbations**	• Physical activity and exercise	• Need for oxygen therapy
**• Smoking status**		• Palliative support
**• BMI**		
**• Device handling and inhalation technique**		
**• Spirometry (to identify patients who are rapid decliners)**		
**• Vaccination status**		

*BMI* Body mass index, *CAT* COPD assessment test, *COPD* Chronic obstructive pulmonary disease, *mMRC* modified Medical Research Council dyspnoea scale

^a^Patients with mild/moderate/severe COPD should be reviewed annually; patients with very severe COPD should be reviewed at least twice a year [186]

#### Personnel

In many countries, specialist nurses play an important role in the management of COPD alongside physicians [189]. Nurse-led COPD clinics in primary care have been shown to reduce exacerbations, hospitalisations and costs [190]. Their role within the COPD disease management team may vary between countries; however, specialist nurses are invaluable in performing spirometry and providing education and advice to improve medication adherence and self-management. Often nurses are also responsible for performing annual patient reviews, thereby relieving the physician’s work burden, but nurses may not be able to easily detect COPD deterioration, distinguish symptoms from other serious comorbidities or adequately control other comorbidities.

Physicians therefore have an overall responsibility for the patient, as well as a more holistic overview of their health conditions. The physician is also responsible for initiating the multidisciplinary team contacts, as pulmonary rehabilitation is an essential part of moderate-to-severe COPD management and should thus be offered to these patients. The point of escalation from primary care to secondary care, such as hospitalisation, varies between countries and healthcare systems. In general, physicians should refer the patient to secondary care if the patient has severe disease at a young age, or is experiencing distressing breathlessness, fatigue, depression and/or anxiety, chronic hypoxia or a rapid decline in lung function [191]. Importantly, regardless of whether the patient is in primary or secondary care, a timely individualised care plan should be made when disease deterioration has reached the level of requiring palliative care.

## Conclusions

COPD is a serious public health burden that is characterised by underdiagnosis, progressive deterioration and treatment difficulties, which all lead to significant disease burden and increased mortality among patients. Correct diagnostic methods are crucial to reduce both under- and overdiagnosis. Besides smoking cessation, physical activity and pulmonary rehabilitation, individualised pharmacological treatment based on symptom burden, exacerbation risk and inflammatory features, such as blood eosinophil count, is central for a favourable prognosis and reduced risk of mortality. Most patients with COPD are fully managed in primary care, presenting unique opportunities to provide patients with evidence-based treatments and individualised care plans. While progress in the correct use of COPD guidelines has been observed recently [8], there is scope for further improvement, particularly in detection rates, disease monitoring/treatment and structured follow-ups [8, 10].

Primary care practitioners need to become aware of the huge impact of COPD (especially in multimorbid patients), have a local support system (e.g. routines, flow charts and nurse-led COPD clinics at the primary healthcare centre), have non-judgemental views of smoking and of COPD as a chronic disease, use a holistic consultation approach and actively motivate the patient to adhere to treatment. Together, these factors could help optimise outcomes for patients with COPD seen in primary care settings.

## Data Availability

Not applicable.

## Abbreviations

CAT: COPD assessment test
COPD: Chronic obstructive pulmonary disease
CVD: Cardiovascular disease
EOS: Eosinophil count
FEV_1_: Forced expiratory volume in 1 second
FVC: Forced vital capacity
GERD: Gastro-oesophageal reflux disease
GOLD: Global Initiative for Chronic Obstructive Lung Disease
HRQL: Health-related quality of life
ICS: Inhaled corticosteroids
LABA: Long-acting β_2_-agonist
LAMA: Long-acting muscarinic antagonist
mMRC: Modified Medical Research Council dyspnoea scale
NT-proBNP: N-terminal pro B-type natriuretic peptide
